# Peer Review of “Detecting Substance Use Disorder Using Social Media Data and the Dark Web: Time- and Knowledge-Aware Study”

**DOI:** 10.2196/58317

**Published:** 2024-05-01

**Authors:** Vetriselvan Subramaniyan

**Affiliations:** 1 Pharmacology Unit, Jeffrey Cheah School of Medicine and Health Sciences, Monash University Malaysia, Sunway City, Malaysia

**Keywords:** opioid, substance use, substance use disorder, social media, US, opioid crisis, mental health, substance misuse, crypto, dark web, users, user perception, fentanyl, synthetic opioids, United States

*This is the peer-review report for “Detecting Substance Use Disorder Using Social Media Data and the Dark Web: Time- and Knowledge-Aware Study.”*

## Round 1 Review

The paper [1] is titled ““Can We Detect Substance Use Disorder?”: Knowledge and Time Aware Classification on Social Media from Darkweb.”

### General Comments

1. The paper has been written comprehensively.
2. The study aims to analyze substance use posts on social media from the dark web and detect substance use disorder using appropriately developed state-of-the-art deep learning and knowledge-aware Bidirectional Encoder Representations From Transformers–based models.
3. Topic analysis is performed to appropriately identify correlations between different drugs and the topics discussed in social media posts.
4. The most effective model achieves statistically significant performance (macro–F_1_-score 82.12, recall 83.58) in accurately identifying substance use disorder.

### Minor Comments

1. The study acknowledges the challenges of crawling crypto markets and the restricted crawling process, which limits the data available for analysis.

Need to explain in the manuscript.

2. The study proposes building an opioid drug social media knowledge graph but does not provide details on the potential impact or implications of such a graph.

Need to provide the details in the manuscript.

3. The study explicitly states that it does not make any clinical diagnosis or treatment suggestions, which indicates a gap in translating the research findings into practical applications for addressing substance use disorder.

Need to justify how this study will be helpful for clinical situations.

### Report

After incorporating the suggested comments, this paper is suitable for publication in the *Journal of Medical Internet Research*.
